# Development, reliability and validity of the Charcot-Marie-Tooth disease Pediatric Scale (CMTPedS)

**DOI:** 10.1186/1757-1146-4-S1-O12

**Published:** 2011-05-20

**Authors:** Joshua Burns, Richard Finkel, Tim Estilow, Andy Hiscock, Matilde Laura, Polly Swingle, Agnes Patzko, Allan Glanzman, Gyula Acsadi, Francesco Muntoni, Mary Reilly, Davide Pareyson, Isabella Moroni, Emanuela Pagliano, Sindhu Ramchandren, Kate Eichinger, Monique Ryan, Robert Ouvrier, Michael Shy, Rosemary Shy

**Affiliations:** 1 Children's Hospital at Westmead, Sydney, Australia; 2 Children's Hospital of Philadelphia, PA, USA; 3 Great Ormond Street Hospital, London, UK; 4 National Hospital for Neurology and Neurosurgery, London, UK; 5 Wayne State University Detroit, MI, USA; 6 Children's Hospital of Michigan, Detroit, MI, USA; 7 C. Besta Neurological Institute, Milan, Italy; 8 University of Rochester, Rochester, NY, USA; 9 Royal Children's Hospital, Melbourne, Australia

## Background

Charcot-Marie-Tooth disease (CMT) causes peripheral nerve demyelination, progressive foot weakness, cavus deformity, difficulty walking and sensory loss. There is a need for accurate, sensitive and disease-relevant measures of young children through to adolescents with CMT to enable accurate assessment of baseline performance, monitor disease severity longitudinally, and determine responses to existing and novel foot and ankle interventions. Our objective was to develop a multidimensional scale to measure disease severity of children with CMT, known as the CMT Pediatric Scale (CMTPedS).

## Methods

The CMTPedS has undergone a thorough development process: (1) definition of the construct; (2) generation of the item pool; (3) choice of scoring format; (4) peer-review (face validity); (5) pilot testing; (6) standardised training; (7) inter-rater reliability of four international centres assessing eight children with CMT; (8) multicenter implementation.

## Results

Findings of the development process: (1) the CMTPedS is a composite scale with broad application to measure disease severity of childhood CMT with eight domains capturing symptoms, foot/ankle involvement, lower limb sensation, hand dexterity/strength, balance, motor function; (2) a large pool of items generated from the literature were reduced based on disease-specificity, functional/patient-relevance, reliability/validity, published norms, test duration and ease of interpretation; (3) items collapsed to 5-point Likert scales using z-scores based on age/gender norms; (4) quality, appropriateness and suitability of items peer-reviewed by 23 expert clinicians/researchers/patient representatives at the 168th European Neuromuscular Centre International Workshop; (5) pilot-tested on four children with CMT to check for administration problems, item instructions, order and duration; (6) clinicians from USA, UK, Italy and Australia trained through workshops, online manual and video resources; (7) all items exhibited good to excellent inter-rater reliability (ICC_2,4_0.78-0.99) (8) a multicenter natural history study of children with all types of CMT aged 3-17 years is underway, with 90 children recruited to date.

## Conclusions

Application and psychometric validation of the CMTPedS continues. We plan to apply the final CMTPedS as the primary outcome in clinical trials of podiatric, pharmacological and surgical interventions.

